# Exploring the Mediating Role of Self-Efficacy in the Relationship Between Caregiver Contribution and Resilience in Inflammatory Bowel Disease

**DOI:** 10.3390/bs15101381

**Published:** 2025-10-11

**Authors:** Mattia Bozzetti, Ilaria Marcomini, Alessio Lo Cascio, Maria Rosaria Magurano, Eleonora Ribaudi, Monica Petralito, Ilaria Milani, Simone Amato, Nicoletta Orgiana, Simone Parello, Pierluigi Puca, Franco Scaldaferri, Marianna Mazza, Giuseppe Marano, Daniele Napolitano

**Affiliations:** 1 Direction of Health Professions, ASST Cremona, 26100 Cremona, Italy; 2 Center for Nursing Research and Innovation, Faculty of Medicine and Surgery, Vita-Salute San Raffaele University, 20132 Milan, Italy; 3 Department of Nursing Research and Management, La Maddalena Cancer Center, 90146 Palermo, Italy; 4 Clinical Psychology Unit, Fondazione Policlinico Gemelli IRCCS, 00168 Rome, Italy; 5 CEMAD—Fondazione Policlinico Gemelli IRCCS, 00168 Rome, Italy; 6 School of Nursing, Azienda Socio-Sanitaria Territoriale Sacco-Fatebenefratelli, 20157 Milan, Italy; 7 School of Nursing, Ospedale San Giuseppe-Gruppo MultiMedica, 20123 Milan, Italy; 8 Cardiac Intensive Care Unit, Heart Transplant Centre and ECMO, Azienda Ospedaliera San Camillo Forlanini, 00152 Rome, Italy; 9 Cut—Fondazione Policlinico Gemelli IRCCS, 00168 Rome, Italy; 10 Dipartimento di Medicina e Chirurgia Traslazionale, Università Cattolica del Sacro Cuore, 00168 Rome, Italy; 11 Department of Neuroscience, Section of Psychiatry, Fondazione Policlinico Universitario Agostino Gemelli IRCCS, 00168 Rome, Italy

**Keywords:** caregiver resilience, inflammatory bowel disease, self-efficacy, caregiver burden, self-care contribution, chronic illness, psychological predictors

## Abstract

Inflammatory bowel disease (IBD) affects not only patients but also their informal caregivers. Enhancing caregiver resilience may improve both caregiver well-being and patient outcomes. This study aimed to assess resilience levels in caregivers of individuals with IBD and identify key psychological and contextual predictors, including caregiver contributions to self-care, self-efficacy, and perceived care load. A multicentre cross-sectional study was conducted across nine IBD outpatient clinics in Italy. Caregiver resilience was measured using the Connor–Davidson Resilience Scale (CD-RISC 25). Additional tools included the Caregiver Contribution to Self-Care of Chronic Illness Inventory (CC-SC-CII) and Caregiver Self-Efficacy in Contributing to Patient Self-Care Scale (CSE-CSC). Robust regression models identified predictors of total resilience and its subdomains. Among 275 caregivers (median age 53; 58.2% female) CD-RISC-25 levels were moderately high (median = 74 [IQR = 65–84]), with no significant differences between those caring for patients with Crohn’s disease or ulcerative colitis. SEM analyses showed that self-efficacy significantly predicted contributions to disease monitoring and management, but not maintenance. In turn, the management dimension was significantly associated with higher levels of resilience across four of five domains (trust, control, acceptance of change, and spirituality), while maintenance was uniquely associated with personal competence. Predictors including education and employment status showed no significant direct or indirect effects on CC-SC-CII. In conclusion, caregiver resilience in IBD is positively associated with self-efficacy and active engagement in disease management. Targeted support strategies may strengthen caregiver resources and promote sustainable care.

## 1. Introduction

Inflammatory bowel disease (IBD) is a comprehensive term encompassing ulcerative colitis (UC) and Crohn’s disease (CD). Both may exhibit analogous symptoms; however, the distinction lies in their anatomical positioning (Rajput et al., 2025). CD can affect any part of the gastrointestinal tract, from the mouth to the anus, and involves transmural inflammation. In contrast, UC is confined to the colon and rectum, with inflammation limited to the mucosal layer.

The global prevalence of IBD is steadily increasing, with some high-income regions reporting more than 600 cases per 100,000 people. In Europe, Western countries have some of the highest prevalence rates in the world, often exceeding 500 cases per 100,000 (Kaplan & Windsor, 2021; Mi et al., 2024).

IBD significantly impacts individuals’ daily life, not only because of physical symptoms such as abdominal pain, rectal bleeding, diarrhea, and fatigue, but also due to the emotional and psychological challenges of managing a chronic condition (Bray et al., 2016). Indeed, individuals with IBD often experience psychological distress and emotional burden that can lead to social withdrawal (Chen et al., 2022), difficulties at work (Youssef et al., 2024), and an overall decline in their quality of life (Knowles et al., 2018). In particular, unlike many other chronic illnesses, IBD is characterized by unpredictable flare-ups, gastrointestinal urgency, and often stigmatizing symptoms, such as incontinence or the need for bowel rest. These unique clinical and psychosocial challenges place an additional burden on caregivers, who may be required to provide flexible, on-demand support in both domestic and clinical contexts (Shukla et al., 2018). This shift highlights the critical role of informal caregivers in helping patients cope with daily challenges that arise from the disease’s unpredictable course (McGilton et al., 2018). An informal caregiver, typically a family member or spouse, assists the care receiver with prescription management, post-operative wound care, and transportation to medical appointments. Particularly when the disease’s condition oscillates between remission and exacerbation, the caregiver may need to address the erratic demands of the illness (Balbinot et al., 2023; Fang et al., 2022; Yuan et al., 2025). However, such a degree of involvement may have detrimental effects, as the burden of caregiving has been shown to compromise caregivers’ psychological well-being and overall quality of life (Yuan et al., 2025; Zand et al., 2020).

The ability of caregivers to manage the challenges associated with supporting individuals with chronic conditions largely depends on the adoption of effective coping strategies and the development of resilience. According to this framework (Szanton et al., 2020; Szanton & Gill, 2010), resilience is defined as the ability to withstand, recover from, or bounce back in terms of both mental and physical health in response to challenging situations. It is seen as the capacity of an individual, family, or community to mitigate the potential negative effects of adversity.

In the context of chronic illness, caregiver resilience is shaped by a range of interrelated factors. For instance, a systematic review found that a caregiver’s level of involvement in the self-care activities of the chronically ill individual is linked to the development of adaptive coping mechanisms (Palacio G et al., 2020). These mechanisms, in turn, influence both the perceived caregiving burden and the psychological and practical resources used to manage it.

### Background

Recent evidence has underscored the importance of understanding the psychological resources that enable caregivers to sustain their role in the context of chronic illnesses such as IBD. Among these, self-efficacy and caregiver contribution to self-care have emerged as pivotal components influencing caregiver adaptation and resilience. Self-efficacy, conceptualized by Bandura (1977) as the belief in one’s ability to execute specific behaviors necessary to produce desired outcomes, has been widely studied in health contexts and is considered a core determinant of how caregivers approach and manage challenges (Schumacher et al., 2008; Northouse et al., 2010). High self-efficacy among caregivers has been linked to improved emotional regulation, lower stress levels, and greater use of adaptive coping strategies (Luszczynska et al., 2005). In IBD, where the disease course is unpredictable and symptoms can be socially stigmatizing, self-efficacy may be particularly critical for managing fluctuating care demands.

In parallel, the caregiver’s contribution to self-care, defined as the set of behaviors aimed at maintaining health, monitoring symptoms, and managing exacerbations on behalf of the patient, has gained increasing attention in chronic care models (Vellone et al., 2020b; Riegel et al., 2012). This contribution is not limited to practical assistance but also includes decision-making support, symptom recognition, and reinforcement of treatment adherence. In the case of IBD, caregivers may play a central role in coordinating medical appointments, assisting with nutritional planning, and monitoring for early signs of disease activity, especially during periods of transition or acute flare-ups (Graff et al., 2009; Artom et al., 2019). Research suggests that when caregivers are actively engaged in supporting patient self-care, they experience a greater sense of control and role fulfillment, which may buffer caregiver burden and promote well-being (Clark et al., 2014; Miller & Dimatteo, 2013). In this conceptual framework, engaging in effective self-care support not only benefits the patient but also fosters a sense of competence and control in caregivers, which may serve as a foundation for building resilience in the face of chronic caregiving demands.

However, the relationship between these elements—contextual characteristics (such as education, occupation, and disease severity), caregiver self-efficacy, contribution to self-care, and resilience—remains underexplored, particularly in the context of IBD. The Middle-Range Theory of Self-Care of Chronic Illness, developed by Riegel et al. (Riegel et al., 2012) and later adapted by researchers such as Vellone et al. (Vellone et al., 2019), provides a conceptual framework to understand these dynamics. This model posits that individual and contextual antecedents influence self-care behaviors and that these, in turn, impact health outcomes such as quality of life, psychological well-being, and resilience. In this framework, caregiver self-efficacy serves as a mediator that enables or limits the caregiver’s ability to engage in adequate self-care support, which in turn influences adaptive outcomes, such as resilience (Palacio G et al., 2020). This study is grounded in the Middle-Range Theory of Self-Care of Chronic Illness, which is itself rooted in Dorothea Orem’s Self-Care Deficit Nursing Theory, providing a conceptual bridge between classical nursing perspectives on self-care and contemporary models of chronic illness management.

Despite growing interest in these constructs, few studies have empirically examined the mediated relationships between caregiver characteristics, self-efficacy, and their downstream effects on both contribution to self-care and resilience. In particular, while resilience has often been studied as an outcome in patients, its development and predictors in informal caregivers, especially those supporting individuals with complex chronic diseases like IBD, remain insufficiently addressed (De Maria et al., 2022; Palacio G et al., 2020; Taylor & Stanton, 2007; You et al., 2024). A better understanding of these interconnections is essential to design targeted interventions that not only support patient outcomes but also sustain the well-being and caregiving capacity of informal caregivers. Given the complex demands of IBD caregiving and the limited empirical evidence on the mechanisms linking contextual factors, caregiver self-efficacy, contribution to self-care, and resilience, there is a clear need to investigate how these elements interact within a comprehensive theoretical model.

**Figure 1:**
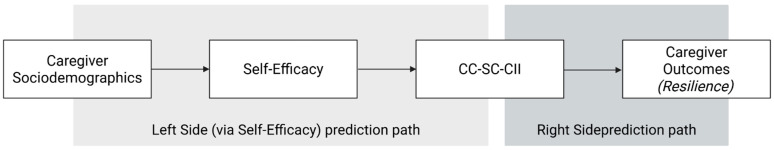
Theoretical model guiding the study. The left side of the model illustrates the hypothesized indirect effects of caregiver sociodemographic and clinical variables on caregiver contribution to self-care via self-efficacy. The right side represents the direct associations between different dimensions of caregiver contribution (maintenance, monitoring, and management) and caregiver resilience outcomes.

Therefore, this study aimed to test the theoretical framework illustrated in Figure 1. Specifically, we hypothesized that sociodemographic and clinical variables would influence caregivers’ contributions to self-care indirectly, through the mediating role of caregiver self-efficacy (left side of the model). Additionally, we examined how the distinct dimensions of caregiver contribution—maintenance, monitoring, and management—predict various aspects of caregiver resilience (right side of the model).

## 2. Materials and Methods

### 2.1. Design

A multicenter, cross-sectional study was conducted between April and June 2024 across nine IBD units in Italy (Napolitano et al., 2024).

### 2.2. Setting and Sampling

A consecutive sample of primary informal caregivers of outpatients diagnosed with IBD was recruited. Patients identified eligible caregivers during routine clinical visits based on their role as the main informal support person involved in daily care and disease management. All eligible caregivers presenting during the study period were invited and enrolled consecutively to minimize selection bias. The study followed the Strengthening the Reporting of Observational Studies in Epidemiology (STROBE) guidelines (von Elm et al., 2014).

### 2.3. Inclusion/Exclusion Criteria

Eligible participants were primary caregivers of adult patients with confirmed diagnosis of CD or UC. Caregivers were required to be 18 years or older and to have provided care for at least six months. To ensure homogeneity, exclusion criteria included caregiving for patients with a disease duration of less than 12 months and those caring for patients with major coexisting chronic illnesses (e.g., cardiovascular disease, diabetes, chronic respiratory diseases, neurodegenerative disorders).

### 2.4. Instruments

A structured questionnaire was developed ad hoc for the purposes of this study. Sociodemographic data were collected through a structured self-report questionnaire administered to all participating caregivers.

The selection of variables was guided by prior research on IBD caregiving and by STROBE recommendations for observational studies. Caregivers reported their age (continuous, in years) and gender. Educational level was initially collected in four categories (primary school, middle school, high school, bachelor’s degree). Because of the limited number of participants with a bachelor’s degree and to ensure adequate cell sizes for the structural equation modeling, education was recoded into a binary variable: low (primary or middle school) versus high (high school or bachelor’s degree). Employment status was first collected as homemaker, retired, student, unemployed, or employed, and later recoded as worker (employed) versus non-worker (all other conditions). Care load was assessed with a single self-report item in which caregivers rated the overall intensity of support they provide. Responses were recorded on a four-point scale (none, mild, moderate, severe) and, for analysis, were grouped as mild, moderate, or severe by combining the “none” and “mild” categories.

Clinical data included the patient’s time since diagnosis, expressed in years as a continuous measure, and current disease activity. Disease activity was assessed using the Mayo score for UC and the Harvey–Bradshaw Index (HBI) for CD. The Mayo score ranges from 0 to 12 and is interpreted as 0–2 = remission, 3–5 = mild, 6–10 = moderate, and ≥10 = severe. The HBI evaluates five parameters—general well-being, abdominal pain, number of liquid stools per day, abdominal mass, and complications—and is interpreted as <5 = remission, 5–7 = mild, 8–16 = moderate, and ≥16 = severe. Biochemical markers (C-reactive protein ≤0.5 mg/dL and faecal calprotectin ≤250 µg/g) collected during routine outpatient visits complemented the clinical indices to provide a comprehensive assessment. Based on these integrated clinical and biochemical data, the treating gastroenterologist classified each patient’s disease activity as remission, mild, moderate, or severe. The original categorical distributions of these variables are reported in the descriptive statistics to preserve full granularity of the sample.

### 2.5. Caregiver Self-Efficacy

Caregiver self-efficacy in supporting patient self-care was measured using the Caregiver Self-Efficacy in Contributing to Patient Self-Care Scale (CSE-CSC) (De Maria et al., 2021), a 10-item instrument adapted from the Self-Care Self-Efficacy Scale (SC-SES) (Yu et al., 2021). It assesses caregivers’ confidence in contributing to the patient’s self-care maintenance, monitoring, and management. Each item is rated on a 5-point Likert scale (1 = not confident to 5 = very confident), with scores standardized on a 0–100 scale—higher scores indicate greater self-efficacy. The scale demonstrates excellent psychometric properties. Exploratory and confirmatory factor analyses supported a two-factor structure (maintenance/monitoring and management) and a second-order hierarchical model (CFI = 0.968, TLI = 0.956, RMSEA = 0.071). Internal consistency was high (Cronbach’s α = 0.94), with composite reliability ranging from 0.90 to 0.91. Construct validity was supported by moderate-to-strong correlations with the Caregiver Contribution to Self-Care of Chronic Illness Inventory (r = 0.45–0.61), and the scale showed good measurement precision (standard error of measurement and smallest detectable change).

### 2.6. Caregiver Contribution to Self-Care

Caregiver involvement in the self-care process was measured using the Caregiver Contribution to Self-Care of Chronic Illness Inventory (CC-SC-CII), a validated 19-item instrument designed to capture the extent of caregiver support across three dimensions of self-care: maintenance, monitoring, and management (Napolitano et al., 2025; Riegel et al., 2012; Vellone et al., 2020a). The self-care maintenance subscale reflects the caregiver’s engagement in promoting the patient’s physical and emotional stability, including efforts to encourage adherence to medication regimens and the adoption of healthy behaviours. The monitoring dimension assesses the caregiver’s contribution to observing the patient’s condition, particularly in identifying symptoms and recognising early changes in health status. The self-care management subscale assesses the caregiver’s role in guiding responses to clinical deterioration, including recognising exacerbations and facilitating appropriate responses. All items are rated on a five-point Likert scale, ranging from “never” to “always.” For each subscale, scores are standardised on a 0 to 100 scale, with higher values reflecting a more substantial caregiver contribution to the respective aspect of the self-care process. Psychometric testing confirmed the instrument’s structural validity and reliability (Vellone et al., 2020a). Confirmatory factor analyses supported a two-factor structure for maintenance and management, and a unidimensional structure for monitoring. A simultaneous CFA of the full scale demonstrated good model fit (CFI = 0.933; RMSEA = 0.055). Internal consistency was excellent (Cronbach’s α = 0.76–0.93), and all subscales showed factor score determinacy coefficients > 0.70 and global reliability indices above the recommended 0.70 threshold for multidimensional constructs.

### 2.7. Resilience Index

To assess resilience levels among participants, the 25-item version of the Connor–Davidson Resilience Scale (CD-RISC 25) was employed, a widely validated instrument originally developed to measure psychological resilience and increasingly utilised in general population studies to monitor changes following therapeutic interventions (Connor & Davidson, 2003; Di Fabio & Palazzeschi, 2012). This instrument, originally developed to measure psychological resilience, has been widely validated and is frequently employed in both clinical and general population studies to monitor changes in response to therapeutic interventions. Rather than offering a unidimensional score, the CD-RISC-25 captures resilience as a multifaceted construct encompassing five interrelated domains. These include spiritual influences, reflecting the role of faith or spiritual beliefs in coping with adversity; personal competence and tenacity, referring to self-confidence, persistence, and the pursuit of high standards; intuitive confidence and emotional regulation, which encompass trust in one’s instincts, the capacity to tolerate distress, and the ability to grow through stress; positive acceptance of change and secure relationships, highlighting adaptability and the presence of supportive social bonds; and finally, a sense of control over life circumstances and events. Each item is rated on a five-point Likert scale ranging from 0 (“not true at all”) to 4 (“true nearly all the time”), yielding a total score between 0 and 100. Higher scores indicate greater levels of resilience. The CD-RISC 25 has demonstrated strong psychometric properties, including internal consistency and sensitivity to change, making it a reliable tool for longitudinal assessments. Authorization for the use of the CD-RISC-25 was obtained directly from the original developers, and the academic licensing fee required for research use was duly paid in accordance with copyright policy.

### 2.8. Statistical Analysis

Descriptive statistics were computed for all study variables prior evaluations of variables distribution (visual residuals distribution, skewness and curtosis). Continuous variables were summarized using medians and interquartile ranges (IQR), while categorical variables were presented as frequencies and percentages.

To examine potential differences in resilience across diagnostic groups, a series of Wilcoxon rank-sum tests were conducted to compare the total CD-RISC-25 score and each of the five resilience dimensions between caregivers of patients with CD and those with UC. This nonparametric approach was selected because the outcome variables exhibited non-normal distributions.

Data were analyzed using structural equation modeling (SEM) in R (version 4.5.0) with the lavaan package. To address the study objectives, two separate models were estimated to reflect the conceptual framework. Model 1 examined the associations between sociodemographic variables (patients’ disease activity, caregiver educational level, caregiver occupational status, age, gender, and patient pathology), CSE-CSC and CC-SC-CII dimensions (Maintenance, Monitoring, and Management). Direct and indirect effects were estimated to evaluate the mediating role of CSE. Model 2 tested the associations between the three CC-SC-CII dimensions and five domains of CDRISK-25 (personal competence, trust, life control, acceptance of change, and spiritual influence). Models fit was assessed using multiple indices, including the Chi-square statistic (χ^2^), the Comparative Fit Index (CFI), the Root Mean Square Error of Approximation (RMSEA) with 90% confidence intervals, and the Standardized Root Mean Square Residual (SRMR) with established cut-offs (Hu & Bentler, 1999). Bias-corrected bootstrap confidence intervals were calculated with 5000 resamples to assess the precision of the effects.

The decision to split the model into two parts was made to maintain an appropriate parameter-to-observation ratio and to ensure that the statistical analysis aligned with the theoretical structure of the conceptual framework. Posterior power estimates based on Bayesian MCMC (Gelman et al., 2013; Holtmann et al., 2016) sampling suggested strong support for most hypothesized pathways. In Model 1, posterior power values for key indirect effects (e.g., disease activity → CSE-CSC → CC-SC-CII) ranged from 0.76 to 0.96. In Model 2, pathways linking CC-SC-CII to CDRISC-25 dimensions showed high posterior power (≥0.84).

In addition to the main predictors, we included age, gender and pathology as covariates in all structural models. These variables were selected based on a directed acyclic graph (DAG) developed using the *dagitty* R package (Textor et al., 2011), which identified them as potential confounders of the relationship between self-efficacy and CC-SC-CII dimensions, as well as the relationship from CC-SC-CII to resilience. The DAG indicated that these variables may influence both the exposure (CSE-CSC) and the outcomes (CC-SC-CII and CDRISC-25) and should therefore be adjusted for to obtain unbiased estimates of the causal effects.

Ordinal predictors (e.g., education, employment) were recoded into binary indicators for SEM to maintain a conservative parameter–to–observation ratio and model stability, given the number of latent variables and paths estimated. The original multi-level distributions are reported in Table 1 for transparency; recoding was applied only to the structural models to reduce overparameterization and sparse cells across covariate-by-path combinations.

#### Sample Size Estimation

To evaluate whether the available sample size was adequate for the planned structural equation models, we performed a posteriori power analysis tailored to SEM. We focused on the RMSEA as the primary index of model fit. For a model of comparable complexity to our final models (approximately 35–40 free parameters), and assuming an α level of 0.05, a null RMSEA of 0.05 (close fit) versus an alternative RMSEA of 0.08, the required sample size to achieve 80% power is approximately 200 participants. Our analytic sample of 275 caregivers therefore exceeds this threshold and provides an estimated power of roughly 0.90 to detect lack-of-fit at the specified RMSEA alternative.

To complement this global fit assessment, we also computed posterior power for key structural paths using Bayesian Markov chain Monte Carlo (MCMC) simulation with 5000 draws (as implemented in the *lavaan* package). These simulations yielded posterior power estimates ranging from 0.76 to 0.96 for the principal hypothesised indirect effects, indicating that the available sample was adequate to detect the expected effect sizes in both models. Taken together, these results support that the achieved sample size offered sufficient statistical power for the planned SEM analyses.

### 2.9. Ethical Consideration

This study was conducted in accordance with the principles of Good Clinical Practice and the revised Declaration of Helsinki. Ethical approval was granted by the Territorial Ethics Committee (Approval ID: 0023486/23) on 2 August 2023, and the study was prospectively registered on ClinicalTrials.gov (Identifier: NCT06015789). Because caregivers were identified through patients attending routine outpatient visits, we recognized the possibility of selection bias and the risk that caregivers might feel obliged to participate. To minimize these risks, patients were only asked whether a primary informal caregiver existed and, if so, invited to provide that person’s contact information. Members of the research team—who were independent of the patients’ attending physicians and not involved in their clinical care—subsequently approached caregivers separately, outside the clinical consultation, explained that participation was entirely voluntary, and emphasized that declining or withdrawing would not affect the patient’s care in any way. All participants received both oral and written information detailing the study’s aims and procedures and provided written informed consent before enrolment. Confidentiality and anonymity were guaranteed, and all data were handled in accordance with applicable data protection regulations.

## 3. Results

### 3.1. General Description

The sample consisted of 275 caregivers, with a median age of 53 years (interquartile range [IQR] = 42.5–59.5) who cared for patients with either CD (n = 150, 54.5%) or UC (n = 125, 45.5%). Socio-demographic characteristics of the sample are reported in Table 1.

Resilience levels were generally moderate-to-high, with comparable scores between those assisting patients with CD and UC. Caregiver self-efficacy was high overall and, in structural models, aligned most closely with behaviors requiring active decision-making (monitoring and management). Of the three caregiver contribution domains, management showed the most consistent links with resilience facets, whereas maintenance related selectively to personal competence, and monitoring showed limited associations.

### 3.2. Contribution to Self-Care and Resilience

The median scores for CC-SC-CII Maintenance, CC-SC-CII monitoring, and CC-SC-CII management were 54.99 [IQR = 36–75], 67.33 [IQR = 50–95], and 56.18 [IQR = 42–75], respectively. CSE-CSC was high, with a median of 71.71 [IQR = 61.25–82.5]. In terms of resilience, scores for CD-RISC 25 are reported in Table 2.

No significant difference was found in spiritual influences (W = 10244, *p* = 0.2145), personal competence (W = 10477, *p* = 0.1108), or acceptance of change (W = 10392, *p* = 0.143). Similarly, trust in one’s intuition (W = 10350, *p* = 0.1623) and life control (W = 10404, *p* = 0.1381) did not significantly differ between the groups. While the total resilience score showed a trend toward higher values in one group, the difference did not reach statistical significance (W = 10574, *p* = 0.0831).

### 3.3. Factors Associated with Resiliency

**Figure 2:**
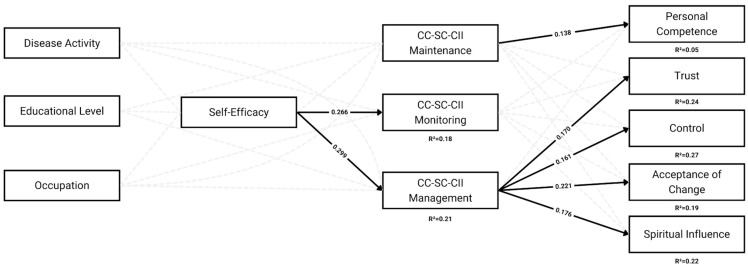
A path diagram combines two distinct structural equation models. Model 1 (Left-side) includes sociodemographic and clinical predictors (Disease Activity, Educational Level, Occupation) influencing Self-Efficacy, which in turn predicts CC-SC-CII Maintenance, Monitoring, and Management. Model 2 (Right-side) examines how these CC-SC-CII dimensions are associated with five facets of resilience (Personal Competence, Trust, Control, Acceptance of Change, and Spiritual Influence). Solid lines represent statistically significant standardized paths (*p* < 0.05), while dashed lines indicate non-significant paths. Coefficients are shown for significant associations.

Path models can be seen in Figure 2.

#### 3.3.1. Model 1: Left Side

CSE-CSC was positively associated with CC-SC-II. Specifically, CSE-CSC significantly predicted Monitoring (β = 0.27, 95% CI [0.28, 0.71], *p* < 0.001) and Management (β = 0.30, 95% CI [0.24, 0.57], *p* < 0.001), but its association with Maintenance was not significant (β = 0.07, 95% CI [−0.07, 0.26], *p* = 0.219).

Pathology was significantly associated with lower levels of CC-SC-CII Maintenance (β = −0.21, *p* = 0.001), with caregivers of patients with CD reporting lower scores compared to those caring for patients with UC, and gender was significantly associated with CC-SC-CII Management, with male caregivers reporting lower scores (β = −0.17, *p* = 0.003). No other covariate effects were statistically significant. The model showed an optimal fit to the data, χ^2^(9) = 16.74, *p* = 0.053, CFI = 0.94, RMSEA = 0.056 (90% CI [0.000, 0.097]), and SRMR = 0.038 (Table 3).

#### 3.3.2. Model 2: Right-Side

Model 2 examined the effects of caregiver self-care dimensions on five aspects of CD-RISC-25. Among the predictors, only the CC-SC-CII Management dimension showed significant associations. Specifically, it was significantly related to Trust (β = 0.17, *p* = 0.013), Life Control (β = 0.16, *p* = 0.018), Acceptance of Change (β = 0.22, *p* = 0.001), and Spiritual Influence (β = 0.18, *p* = 0.006). Maintenance was positively associated only with Personal Competence (β = 0.14, *p* = 0.046), while Monitoring was not significantly associated with any resilience outcomes (all *p* > 0.5). The total standardized effects of all three CC-SC-CII dimensions on the five resilience domains ranged from β = 0.23 to β = 0.27, all statistically significant (*p* < 0.01). Gender was significantly associated with trust (β = 0.12), indicating that male caregivers reported higher levels of trust compared to female caregivers. The model showed an optimal fit to the data, χ^2^_(30)_ = 38.42, *p* = 0.133, CFI = 0.97, RMSEA = 0.038 (90% CI [0.000, 0.068]), and SRMR = 0.034 (Table 4).

## 4. Discussion

This study aimed to explore the mediating role of caregiver self-efficacy in the relationship between sociodemographic/clinical factors and caregiver contributions to self-care, and to assess the effect of these contributions on caregiver resilience in the context of IBD. The findings of this study reveal a differentiated role of caregiver self-efficacy across the three domains of caregiver contribution to self-care. Specifically, self-efficacy was significantly associated with monitoring and management behaviors, but not with maintenance activities. This suggests that caregivers’ confidence in their ability to provide support is most critical when it comes to behaviors that demand proactive engagement, decision-making, and emotional regulation, such as symptom observation, response to flare-ups, or coordination of medical care. These findings are consistent with Bandura’s social cognitive theory, which posits that self-efficacy exerts the greatest influence on behaviors perceived as complex, novel, or emotionally demanding. Similarly, the Middle-Range Theory of Self-Care for Chronic Illness highlights that self-efficacy facilitates complex self-care tasks that require judgment, adaptation, and problem-solving (Bandura, 1997; Riegel et al., 2012).

By contrast, maintenance behaviors, which refer to routine support such as medication reminders, diet supervision, or daily assistance, appear to be less dependent on perceived self-efficacy. This may be because such actions are often habitual, context-driven, and reinforced by established caregiving routines. Previous research has suggested that low-complexity or repetitive tasks may rely more on external motivators or contextual expectations than on internal beliefs of efficacy (Sebern & Riegel, 2009). Unexpectedly, none of the sociodemographic or clinical characteristics (e.g., caregiver educational level, employment status, or patient’s disease activity) emerged as significant predictors of self-efficacy or indirect contributors to resilience through the mediation model. This diverges from findings in other caregiving contexts, such as heart failure, cancer, or dementia, where higher caregiver education and care experience have been associated with greater perceived self-efficacy (Grano et al., 2017; Chung et al., 2010). One possible explanation is that caregiving in IBD is uniquely shaped by disease unpredictability and psychosocial factors, such as embarrassment, uncertainty, or stigma, which may moderate the impact of structural characteristics. In such conditions, psychological resources, family dynamics, and access to professional support may be more salient than background demographics.

In the second part of the model, the management dimension of caregiver contribution to self-care was found to be the most potent predictor of resilience, with significant associations observed across four of five domains of the CD-RISC-25: “Acceptance of Change”, “Spiritual Influence”, “Trust”, and “Life Control”. These findings support prior research indicating that active illness management enhances caregiver adaptation by reinforcing self-worth, a sense of purpose, and perceived mastery. Such active engagement may serve as a psychological anchor for caregivers, reinforcing identity, emotional resilience, and long-term coping capacity. Similarly, recent evidence suggests that resilience also plays a critical role in mitigating psychological distress among individuals with IBD themselves, particularly in buffering symptoms of depression and anxiety (Ferrarese et al., 2025). This parallel reinforces the dyadic relevance of resilience in the IBD context, affecting both caregivers and patients. In contrast, the monitoring dimension, although theoretically linked to vigilance and symptom awareness, showed no significant associations with resilience. This may be because passive observation in the absence of meaningful action could generate worry or hypervigilance, potentially exacerbating distress rather than promoting adaptation (Chang et al., 2023; Vitaliano et al., 2003).

Interestingly, the maintenance dimension was uniquely associated with personal competence, suggesting that consistent, routine caregiving behaviors—such as assisting with daily activities or adhering to medication regimens—may reinforce caregivers’ beliefs in their stability, dependability, and endurance, which are core facets of resilience (Connor & Davidson, 2003). These results align well with Model 1, where self-efficacy influenced engagement in specific caregiving behaviors. Taken together, the two parts of the model suggest that psychological beliefs translate into behavioral engagement, which in turn supports the development of resilience (Dionne-Odom et al., 2021).

However, these behaviors may lack the novelty, variability, or problem-solving demand required to influence more dynamic aspects of resilience (e.g., creativity, adaptability).

Taken together, these findings validate a mediated pathway in which caregiver self-efficacy enables certain self-care behaviors (particularly management and monitoring), which in turn enhance specific resilience resources. This model echoes the Middle-Range Theory of Self-Care reinforcing the idea that psychological traits (such as self-efficacy) are proximal drivers of caregiving actions, which ultimately shape adaptive outcomes (Riegel et al., 2012; Vellone et al., 2020b). Notably, the use of SEM offered a valuable framework for simultaneously testing direct and indirect effects, providing a more integrated and dynamic understanding of the relationships between self-efficacy, caregiving behaviors, and resilience.

Moreover, the differential effects across self-care dimensions and resilience domains point to the need for nuanced interventions: while coaching and empowerment may bolster efficacy and complex caregiving skills (e.g., crisis response), routine-oriented training or environmental support may be more effective in sustaining daily maintenance behaviors. These findings highlight that one-size-fits-all caregiver programs may fall short, and that tailored approaches addressing the psychological and behavioral specificities of IBD caregiving are essential for optimizing both caregiver and patient outcomes (Meyer et al., 2022).

In this context, the integration of multidisciplinary care teams, including IBD nurses (Schiavoni et al., 2025; Kemp et al., 2018), psychologists, social workers, nutritionists, and gastroenterologists, becomes crucial. Such teams can collaboratively address the multifaceted needs of both patients and caregivers by combining clinical expertise with psychosocial support and educational interventions (Fiorino et al., 2025). For example, nurses and psychologists can deliver targeted interventions to strengthen caregiver self-efficacy and stress management. At the same time, dietitians and physicians can help caregivers understand and manage dietary and medical regimens. This interprofessional collaboration ensures continuity of care, facilitates the personalization of support strategies, and enhances the overall effectiveness of interventions aimed at improving caregiver resilience and well-being (D’Onofrio et al., 2025).

### 4.1. Strengths and Limitations

This study presents several strengths. It is among the first to empirically examine the mediated role of caregiver self-efficacy in shaping contributions to self-care and the downstream impact on multiple dimensions of resilience in the context of IBD caregiving. The use of validated instruments and a multicenter design enhances the reliability of the findings and their applicability across varied clinical contexts. Moreover, the differentiation of self-care behaviours into maintenance, monitoring, and management provides a nuanced understanding of caregiving dynamics and allows for targeted interpretation of the results. Recruitment through patient referral may have introduced a selection bias by favouring caregivers who were present at clinic visits or who maintain close contact with the patient. Although every effort was made to guarantee voluntary participation and to prevent any perception of coercion, we cannot exclude the possibility that more disengaged caregivers were under-represented.

However, several limitations should be acknowledged. First, the cross-sectional design limits causal inference; future longitudinal investigations are required to confirm the directionality and stability of the observed relationships and to explore potential reciprocal effects between caregiver self-efficacy, contribution to self-care, and resilience over time. Second, although we describe our recruitment as convenience sampling, in practice, it followed a consecutive sampling procedure in which all eligible caregivers attending outpatient clinics during the study period were invited to participate. While this approach reduces selection bias compared with simple convenience sampling, it still relies on patient referral. It may not capture caregivers who were less engaged or unable to accompany patients, thereby limiting generalizability.

Third, all measures were obtained through self-report questionnaires, which introduces the possibility of recall bias and social desirability bias; responses may therefore not fully reflect actual behaviours or psychological states. Fourth, the absence of significant associations between specific predictors and the CC-SC-CII may in part reflect our analytic choices. Education and employment status were treated as binary indicators to ensure model parsimony and stability in structural equation modelling. While small cell sizes drove this decision, it inevitably entailed trade-offs: dichotomization can reduce variability and statistical power, obscure non-linear or threshold relationships, leave residual confounding within collapsed strata, and limit generalizability when meaningful distinctions—such as between high school and bachelor’s degree—are masked.

Fifth, the path model was divided into two components (left and right sides) as a methodological compromise. A single integrated model would have allowed for the simultaneous testing of all hypothesized direct and indirect effects. Still, our sample size did not meet the recommended thresholds for such complex structural models, particularly given the number of estimated parameters. Future studies with larger samples aim to validate the complete model within a unified analytic framework.

Finally, we were unable to assess certain potentially relevant variables—such as individual coping style, the level of emotional support received, and the quality of the caregiver–patient relationship—which might influence both self-efficacy and resilience. These factors, along with the cross-sectional design, consecutive but clinic-based sampling, and reliance on self-report, should be considered when interpreting our findings and planning future research.

### 4.2. Clinical and Practical Implications

The findings of this study have important implications for clinical practice and caregiver support programs.

First, the demonstrated mediating role of self-efficacy indicates that strengthening caregivers’ confidence in their ability to provide care is not only beneficial for daily disease management but also essential for enhancing psychological resilience. This highlights the need for interventions that build self-efficacy, such as individualized coaching, structured psychoeducational programs, and skills-based workshops tailored to the context of IBD caregiving.

Second, the differential impact of the three self-care dimensions on resilience suggests that interventions should be tailored to specific behaviors. For example, management-related tasks—closely linked to problem-solving and adaptation—may benefit from targeted training in crisis recognition and decision-making. In contrast, maintenance tasks may respond better to environmental restructuring, scheduling tools, and routine reinforcement.

Ultimately, these strategies should be implemented within a multidisciplinary framework that leverages the complementary roles of IBD nurses, psychologists, and social workers. Such an approach ensures caregivers receive personalized, continuous support that addresses not only the medical demands of IBD but also the emotional, cognitive, and practical challenges of long-term caregiving.

## 5. Conclusions

This study supports a mediated model in which caregiver self-efficacy influences specific self-care behaviors, especially management and monitoring, which in turn enhance caregiver resilience. Management behaviors were most strongly associated with key resilience domains, while maintenance had a limited impact. The findings emphasize the importance of behavior-specific, psychologically informed interventions to strengthen caregiver adaptation. The absence of significant sociodemographic predictors suggests that internal psychological resources may be more crucial than structural characteristics. Finally, integrating these insights into multidisciplinary care can enhance the well-being of both caregivers and patients, improving the overall quality of IBD care.

## Figures and Tables

**Table 1:** Sociodemographic characteristics of the sample.

Variables	CD (n = 131, 48.4%)	UC (n = 144, 51.6%)
**Age** Me [IQR]	50 [40.5–59]	53.5 [44–60]
**Gender** n (%)		
Female	76 (58%)	84 (58.3%)
Male	55 (42%)	60 (41.7%)
**Care Load** n (%)		
Mild	54 (41.2%)	49 (34%)
Moderate	60 (45.8%)	76 (52.8%)
Severe	17 (13%)	18 (12.5%)
**Educational Level** n (%)		
Bachelors	29 (22.1%)	44 (30.6%)
High School	66 (50.4%)	68 (47.2%)
Middle School	33 (25.2%)	28 (19.4%)
Primary School	3 (2.3%)	4 (2.8%)
**Work Status** n (%)		
Homemaker	15 (11.5%)	14 (9.7%)
Retired	21 (16%)	25 (17.4%)
Student	6 (4.6%)	2 (1.4%)
Unemployed	6 (4.6%)	2 (1.4%)
Worker	83 (63.4%)	101 (70.1%)
**Relationship** n (%)		
Partner/Spouse	63 (48.1%)	84 (58.3%)
Family (Parent/Sibling)	26 (19.8%)	24 (16.7%)
Other Family	12 (9.2%)	14 (9.7%)
Friend	18 (13.7%)	11 (7.6%)
No relationship specified	12 (9.2%)	11 (7.6%)
**Patients Time from diagnosis** (y) M(SD)	11.2 (9.96)	11.5 (8.74)
**Patients Disease Activity** n (%)		
Remission	98 (74.8%)	51 (35.4%)
Mild	14 (10.7%)	24 (16.7%)
Moderate	12 (9.2%)	34 (23.6%)
Severe	7 (5.3%)	35 (24.3%)
**Time in Charge** n (%)		
<1 Year	29 (22.1%)	35 (24.3%)
1–3 Year	21 (16%)	29 (20.1%)
3–5 Years	18 (13.7%)	22 (15.3%)
>5 Years	63 (48.1%)	58 (40.3%)

Note: CD: Crohn’s Disease; UC: Ulcerative Colitis.

**Table 2:** Resiliency scores across pathologies.

Resilience Domains	Entire Sample (n = 275)	CD (n = 131)	UC (n = 144)	*p*-Value ^§^
Spiritual Influences	8 [6–10]	8 [6–10]	8 [6–10]	0.214
Personal Competence	15 [13–18.5]	16 [14–19]	15 [13–18]	0.110
Acceptance of Change	15 [13–18]	16 [13–18]	15 [13–17.2]	0.143
Trust in one’s intuition	21 [18–24]	21 [18–24]	21 [17–24]	0.162
Life Control	15 [12–18]	15 [13–18]	15 [12–17]	0.138
Resiliency (Total Score)	74 [65–84]	76 [65.5–85]	72 [64–83]	0.083

Note: CD: Crohn’s Disease; UC: Ulcerative Colitis. ^§^ Wilcoxon Tests.

**Table 3:** Standardized direct and indirect effects of predictors and caregiver self-efficacy on caregiver contribution to self-care dimensions. Indirect effects represent mediation via self-efficacy.

Pathway	CC-SC-CII Maintenance	CC-SC-CII Monitoring	CC-SC-CII Management
	Β [IC95%]	Β [IC95%]	Β [IC95%]
**Indirect Effects** (via *Self-Efficacy*)			
Disease Activity → CSE-CSC → CC-SC-CII	0.002 [−0.155, 0.351]	0.009 [−0.596, 1.184]	0.010 [−0.479, 0.941]
Educational Level → CSE-CSC → CC-SC-CII	−0.001 [−0.769, 0.668]	−0.003 [−2.611, 2.477]	−0.004 [−2.104, 1.952]
Occupational Status → CSE-CSC → CC-SC-CII	0.003 [−0.446, 0.932]	0.012 [−1.656, 3.286]	0.013 [−1.190, 2.822]
**Direct Effects**			
CSE-CSC → CC-SC-CII	0.069 [−0.069, 0.264]	**0.266 [0.277, 0.712]**	**0.299 [0.244, 0.571]**
Disease Activity → CC-SC-CII	0.034 [−1.134, 2.206]	0.034 [−1.134, 2.206]	0.034 [−1.134, 2.206]
Educational Level → CC-SC-CII	−0.013 [−5.175, 4.344]	−0.013 [−5.175, 4.344]	−0.013 [−5.175, 4.344]
Occupational Status → CC-SC-CII	0.044 [−3.056, 6.288]	0.044 [−3.056, 6.288]	0.044 [−3.056, 6.288]

Notes: Betas (β) are fully standardized. Bold values are statistically significant paths. CSE-CSC = Self Efficacy; CC-SC-CII = Caregiver Contribution to Self-Care Chronic Illness Inventory. Bold values indicate statistically significant results (*p* < 0.05).

**Table 4:** Standardized path coefficients from CC-SC-CII dimensions (Maintenance, Monitoring, Management) to Resilience outcomes (Personal Competence, Trust, Life Control, Acceptance of Change, Spiritual Influence).

Predictors	Personal Competence β [IC95%]	Trust β [IC95%]	Life Control β [IC95%]	Change Acceptance β [IC95%]	Spiritual Influence β [IC95%]
CC-SC-CII Maintenance	**0.138 [0.001, 0.037]**	0.063 [−0.011, 0.033]	0.106 [−0.003, 0.030]	0.037 [−0.010, 0.020]	0.011 [−0.013, 0.015]
CC-SC-CII Monitoring	−0.029 [−0.019, 0.013]	0.029 [−0.017, 0.024]	−0.043 [−0.019, 0.010]	0.015 [−0.013, 0.016]	0.069 [−0.007, 0.019]
CC-SC-CII Management	0.117 [−0.001, 0.037]	**0.170 [0.008, 0.061]**	**0.161 [0.004, 0.044]**	**0.221 [0.014, 0.049]**	**0.176 [0.006, 0.035]**

Notes: Betas (β) are fully standardized. Bold values are statistically significant paths. CC-SC-CII = Caregiver Contribution to Self-Care Chronic Illness Inventory. Bold values indicate statistically significant results (*p* < 0.05).

## Data Availability

The data presented in this study are available on request from the corresponding authors.

## Abbreviations

The following abbreviations are used in this manuscript:

IBD	Inflammatory Bowel Disease
CD	Crohn’s Disease
UC	Ulcerative Colitis
CC-SC-CII	Caregiver Contribution to Self-Care of Chronic Illness Inventory
CD-RISC-25	Connor–Davidson Resilience Scale—25 items
CSE-CSC	Contributing to Patient Self-Care Scale
IQR	Interquartile Range
SE	Standard Error
STROBE	Strengthening the Reporting of Observational Studies in Epidemiology
IRB	Institutional Review Board
